# A case report: Neuropsychiatric presentation of atypical Rasmussen’s encephalitis

**DOI:** 10.4102/sajpsychiatry.v32i0.2523

**Published:** 2026-08-12

**Authors:** Johanna H.C. Landman, Sibusiso Sotobe-Mose

**Affiliations:** 1 Department of Psychiatry, School of Clinical Medicine, Faculty of Health Sciences, University of the Witwatersrand, Johannesburg, South Africa

**Keywords:** Rasmussen’s encephalitis, neuropsychiatry, adult-onset epilepsy, major neurocognitive disorder, treatment resistant epilepsy

## Abstract

**Introduction:**

Rasmussen’s encephalitis (RE) is a rare, progressive inflammatory neurological disorder characterized by treatment-resistant epilepsy, progressive hemiplegia, and cognitive decline. Although its pathogenesis remains incompletely understood, immune-mediated mechanisms are thought to play a central role. RE predominantly affects children, with only approximately 10% of cases occurring in adults. Diagnosis relies on the combination of clinical features, serial neuroimaging, and electroencephalographic findings. Early recognition is essential, as treatment options are limited and outcomes are improved with timely intervention. Atypical adult-onset presentations are particularly uncommon and may present significant diagnostic challenges.

**Patient presentation:**

We report a case that presented with atypical, adult-onset RE with major neurocognitive disorder.

**Management and outcome:**

The patient’s diagnosis was made with neuroimaging 10 years following seizure onset. Findings indicated moderate generalised atrophy, with asymmetric involvement of the left cerebral hemisphere. Interventions required a multidisciplinary team, and pharmacological management included sodium valproate and lamotrigine for seizure control, as well as quetiapine and fluoxetine for behavioural disturbances. The patient’s prognosis remains poor because of generalised brain atrophy limiting treatment options.

**Conclusion:**

There is a need to raise awareness of RE to assist with early detection and treatment. Late diagnosis may have contributed to the patient’s poor prognosis.

**Contribution:**

Despite the need for further research, early neuroimaging of patients with new adult-onset seizures may help decrease morbidity associated with RE.

## Introduction

Rasmussen’s encephalitis (RE) is a rare epilepsy syndrome associated with treatment-resistant seizures, cognitive decline and developmental sequelae.^1,2,3,4,5,6^ Its incidence ranges between 1.7 and 2.4 per 10 million individuals.^2,3,6^ Disease progression typically occurs in three clinical stages:^6^

- Prodromal phase: contralateral hemiparesis and low-frequency focal motor seizures.
- Acute phase: frequent seizures and aphasia if the dominant hemisphere is involved.
- Residual stage: less frequent seizures and irreversible cognitive decline.

Neuroimaging can detect pathological changes from 4 months after disease onset.^7^ Atypical forms of RE include adult-onset, bilateral RE and RE associated with other diseases.^1,8,9^ Adult-onset RE may present as a ‘myoclonic phenotype’ (focal cortical myoclonus) or an ‘epileptic phenotype’ (focal motor seizures and more severe brain atrophy).^1^

The pathogenesis of RE is poorly understood; however, it is postulated to be immune-mediated, involving both adaptive and innate immunity.^1,4,7^ Hemispherectomy remains the only cure for resistant epilepsy, but carries risks of severe postoperative complications.^2,3,4^ During early stages, immunotherapy may slow early progression, but does not prevent cognitive decline; evidence is limited to case series and lacks randomised controlled trials.^4,10^

This case highlights severe cognitive decline in atypical RE and underscores the role of early neuroimaging in reducing morbidity.

## Ethical considerations

Ethical clearance to conduct this study was obtained from the University of the Witwatersrand Human Research Ethics Committee (Ref. No. M241083). Written informed consent for publication was obtained from the patient’s legal guardian.

## Patient presentation

Mr X is a 34-year-old African male who was working as a security guard. He has no history of childhood seizures, head injury, substance use, metabolic disease, human immunodeficiency virus (HIV) or syphilis, and has no significant family history. He was diagnosed with epilepsy at age 24 years, following his first seizure; no neuroimaging was performed at the time. He subsequently developed frequent and severe generalised tonic-clonic seizures, which led to job loss and multiple hospital admissions. Subsequently, he developed a decline in cognitive functioning, including poor attention, memory, executive functioning and social cognition. Over the years, he received valproate, lamotrigine, risperidone, quetiapine and fluphenazine decanoate for epilepsy and behavioural disturbances. Despite medication adherence, he was readmitted several times as a result of uncontrolled seizures and behavioural problems. In 2014, his cognitive decline worsened, rendering him childlike and causing him to struggle with simple tasks, such that Mr X needed constant supervision in performing basic activities of daily living. In 2019, he developed transient hemiplegia, expressive aphasia and agraphia, along with disinhibited behaviour leading to recurrent hospitalisation. Over subsequent admissions, he exhibited severe disorganisation, poor self-care, hoarding behaviour and aggression; clozapine was trialled but discontinued because of seizure risk and poor response.

Mr X was transferred to Sterkfontein Hospital (SFH) because of ongoing problematic behaviour. At SFH, his medication included therapeutic dosages of amisulpride, sodium valproate, lamotrigine, orphenadrine and clothiapine (for sedation when necessary). Neurological examination revealed global aphasia with a positive grasp reflex. He had no cranial nerve fallout. His power was normal in all limbs, but reflexes were reduced. His sensation was decreased globally, and he had markedly reduced pain perception. There were no abnormalities detected on the cerebellum examination. Mental state examination indicated disorientation, incoherence and perseveration. His mood was euthymic with a reactive affect. Formal cognitive testing was limited by the severity of the impairment, with deficits evident in attention, executive function and language.

Mr X cannot fully participate in therapy because of cognitive impairment; however, psychology instituted a basic behavioural plan. Efforts to optimise the dose of amisulpride for behaviour control failed because of hyperprolactinaemia (55.20 µg/L), as did a trial of lithium. The lamotrigine dose was optimised to 150 mg twice a day. Despite initiating quetiapine and optimising to 400 mg twice daily, his behaviour remained unchanged. Fluoxetine was initiated, leading to improvement in impulsive and hoarding behaviour. His seizures remain well controlled on treatment.

**FIGURE 1:**
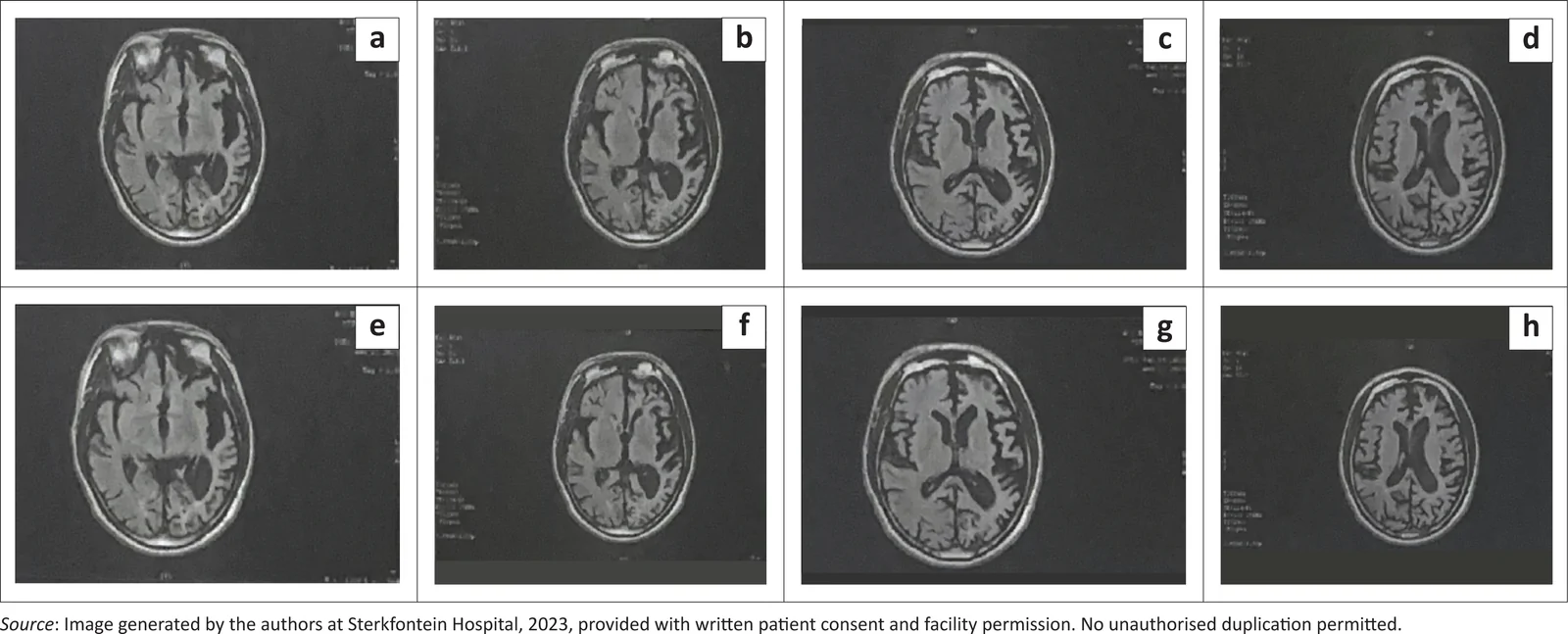
Mr X’s T2 FLAIR axial MRI images (a–h). Significant generalised atrophy, with asymmetric involvement of the left cerebral hemisphere (a–h).

Investigations done included a routine electroencephalogram (EEG) (10 August 2023), which was normal, blood and cerebrospinal fluid analysis, which showed no significant findings (Table 1). His Magnetic Resonance Imaging (MRI) brain (28 August 2023; Figure 1) displayed features consistent with RE of the left cerebral hemisphere, including cortical atrophy and ventricular dilation on a background of generalised brain atrophy. There was no evidence of ongoing inflammation, ischaemia or infarcts.

**TABLE 1:** Investigations and results.

Investigation	Result
Blood results (June 2023 to September 2023)	Full blood count: white cell count 5.57 × 10^9^/L; Hb (g/dL) 16.1; Platelets 258 × 10^9^/L; neutrophils 1.99 × 10^9^/L Renal functions and electrolytes: Sodium (mmol/L) 136; potassium (mmol/L) 5.1; Urea (mmol/L) 3.9; Creatinine (µmol/L) 107 eGFR (mL/min/1.73 m^2^) > 60 Liver function tests: Total protein (g/L) 74; Albumin (g/L) 46; total bilirubin (µmol/L)5; ALT (U/L) 11; AST (U/L) 23; ALP (U/L) 88; GGT (U/L) 50 Inflammatory markers: ESR (mm/h) 3; CRP (mg/L) 1 Metabolic screen: HBA1C 5.6%; Triglycerides (mmol/L) 0.75; Total cholesterol (mmol/L) 3.92; HDL cholesterol (mmol/L) 1.64; LDL cholesterol (mmol/L) 1.64 Thyroid function tests: Thyroid stimulating hormone (mIU/L) 7.21 (elevated); Free T4 (pmol/L) 15.8 (normal) Infection screen: HIV Elisa: negative Treponema pallidum haemagglutination assay (TPHA) positive; rapid plasma regain (RPR) negative Other: Prolactin (µg/L) 55.20 (raised) Ammonia (µmol/L) 29 Rheumatoid factor (IU/mL) 15 (normal) Antinuclear antibodies: negative Compliment C3 (g/L) 1.11 (normal) Compliment C4 (g/L) 0.21 (normal)
Lumbar puncture (11 August 2023)	Colour clear; protein (mg/dL) 0.21; glucose (mg/dL) 3.4; neutrophils (cells/µL) 0, lymphocytes (cells/µL) 0, Red blood cells (cells/µL) 0, Gram stain negative; Cryptococcal antigen negative; venereal disease research laboratory (VDRL) negative; Tuberculosis negative; Indian Ink stain negative
EEG (10 August 2023)	11 Hertz – 12 Hertz, alpha posterior dominant reactive rhythm. No focal slowing. Conclusion: normal EEG

eGFR, estimated glomerular filtration rate; ALT, alanine aminotransferase; AST, aspartate aminotransferase; ALP, alkaline phosphatase; GGT, gamma glutamyl transferase; ESR, erythrocyte sedimentation rate; CRP, c-reactive protein; LDL, low-density lipoprotein; HDL, high-density lipoprotein; HBA1C, glycated haemoglobin; HIV, human immunodeficiency virus; EEG, electroencephalogram.

He was assessed as having Rasmussen encephalitis, currently in the residual phase, with major neurocognitive disorder probable due to RE, with behavioural problems.

Mr X’s prognosis is poor as a result of extensive cerebral atrophy and disease progression limiting treatment options.

## Discussion

Atypical RE is a rare variant of classic childhood-onset, unihemispheric form.^1^ It includes adult-onset disease, bilateral involvement or association with other conditions.^1^ These atypical features often delay diagnosis and limit treatment, as hemispherectomy is usually not feasible, leaving immunotherapy and supportive care as the main options.^2,3,4,10^

The case presented demonstrates such one variant, in which delayed diagnosis contributed to loss of independence and functional decline, resulting in institutionalisation. Diagnosis is also challenging because of broad differentials, which can cloud the clinical picture. Further research is needed to clarify diagnostic pathways and treatment options, including well-designed randomised controlled trials.

## Conclusion

Clinicians should consider atypical RE in individuals presenting with new-onset seizures and cognitive decline, especially in the context of comorbidities, older age and bilateral hemispheric involvement. Early detection and treatment may improve outcomes and reduce morbidity and mortality.
